# Supplementary material for: Concurrence of stunting and overweight/obesity among children: Evidence from Ethiopia
Source: PLoS One. 2021 Jan 15;16(1):e0245456. doi: 10.1371/journal.pone.0245456 (PMC7810347; doi:10.1371/journal.pone.0245456)
Supplement: Attachment — Submitted filename: PONE-D-20-03785_reviewer.pdf [file pone.0245456.s002.pdf]

PLOS ONE

Concurrent of stunting and overweight/obesity among children:evidence from Ethiopia

--Manuscript Draft--

Manuscript Number:	PONE-D-20-03785
Article Type:	Research Article
Full Title:	Concurrent of stunting and overweight/obesity among children:evidence from Ethiopia
Short Title:	Concurrent of stunting and overweight/obesity
Corresponding Author:	Alinoor Mohamed Farah, MPH<br>Jigjiga University<br>Jigjiga, ETHIOPIA
Keywords:	Young children, Concurrent of Stunting and Overweight/Obesity, Stunting, Overweight, DHS, Ethiopia
Abstract:	Background: Nutrition transition in many low- and middle-income countries (LMICs) has led to childhood nutritional outcomes to shift from a predominance of undernutrition to a dual burden of under- and overnutrition. Yet, Infant and young child feeding programs in Ethiopia mainly focus on undernutrition. It is therefore crucial to assess the prevalence and determinants to better inform infant young child feeding programs.<br>Methods: We analyzed anthropometric, sociodemographic and dietary data of children aged 6-23 months from 2016 Ethiopian Demographic and Health Survey (EDHS). A total of 2,674 children were included in the current study. Concurrent of Stunting and Overweight/Obesity (CSO) prevalence was estimated by distal, intermediate and proximal factors. To identify factors associated with CSO, we conducted hierarchical logistic regression analyses.<br>Results: The overall prevalence of CSO was 2.45%. The odds of CSO was significantly higher in children of low household wealth category, compared to their counter parts in the richest category (AOR=2.07, 95%CI=1.06–4.03, P=0.033). In boys, the likelihood of CSO was 1.60 times that of girls (95%CI =1.03–2.49, P =0.038). Above 12months of age was significantly associated with a higher odd of CSO, compared to below 12months of age (AOR=1.76, 95% CI=1.07– 2.88, P=0.026).<br>Small birth size was significantly associated with a higher odd of CSO, compared to large birth size (AOR=2.53, 95%CI=1.45–4.41, P=0.001). Children who did not take vitamin A supplement within the previous six months had a higher odd of CSO, compared to those who took (AOR=1.91, 95%CI =1.19–3.07, P =0.007).<br>Conclusion: Our study provided evidence on the co-existence of stunting and overweight/obesity among infants and young children in Ethiopia. CSO was associated with various factors originating from community and child levels. Therefore, identifying children at risk of growth flattering and excess weight gain provides IYCF programs in Ethiopia and beyond with an opportunity of earlier interventions.
Order of Authors:	Alinoor Mohamed Farah, MPH
	Tahir Yousuf Nour
	Bilal Shikur Endris
	Seifu Hagos Gebreyesus
Additional Information:	
**Question**	**Response**
**Financial Disclosure**<br><br>Enter a financial disclosure statement that describes the sources of funding for the work included in this submission. Review the submission guidelines for detailed requirements. View published research	The author(s) received no specific funding for this work.

Powered by Editorial Manager® and ProduXion Manager® from Aries Systems Corporation

articles from *PLOS ONE* for specific examples.<br><br>This statement is required for submission and **will appear in the published article** if the submission is accepted. Please make sure it is accurate.<br><br>**Unfunded studies**<br>Enter: *The author(s) received no specific funding for this work.*<br><br>**Funded studies**<br>Enter a statement with the following details:<br>• Initials of the authors who received each award<br>• Grant numbers awarded to each author<br>• The full name of each funder<br>• URL of each funder website<br>• Did the sponsors or funders play any role in the study design, data collection and analysis, decision to publish, or preparation of the manuscript?<br>• **NO** - Include this sentence at the end of your statement: *The funders had no role in study design, data collection and analysis, decision to publish, or preparation of the manuscript.*<br>• **YES** - Specify the role(s) played.<br><br>* typeset	
**Competing Interests**<br><br>Use the instructions below to enter a competing interest statement for this submission. On behalf of all authors, disclose any competing interests that could be perceived to bias this work—acknowledging all financial support and any other relevant financial or non-financial competing interests.<br><br>This statement **will appear in the published article** if the submission is accepted. Please make sure it is accurate. View published research articles from *PLOS ONE* for specific examples.	The authors have declared that no competing interests exist.

**NO authors have competing interests**<br><br>Enter: *The authors have declared that no competing interests exist.*<br><br>**Authors with competing interests**<br><br>Enter competing interest details beginning with this statement:<br><br>*I have read the journal's policy and the authors of this manuscript have the following competing interests: [insert competing interests here]*<br><br>* typeset	
**Ethics Statement**<br><br>Enter an ethics statement for this submission. This statement is required if the study involved:<br><br>• Human participants<br>• Human specimens or tissue<br>• Vertebrate animals or cephalopods<br>• Vertebrate embryos or tissues<br>• Field research<br><br>Write "N/A" if the submission does not require an ethics statement.<br><br>General guidance is provided below. Consult the submission guidelines for detailed instructions. **Make sure that all information entered here is included in the Methods section of the manuscript.**	N/A

**Format for specific study types**<br><br>**Human Subject Research (involving human participants and/or tissue)**<br>• Give the name of the institutional review board or ethics committee that approved the study<br>• Include the approval number and/or a statement indicating approval of this research<br>• Indicate the form of consent obtained (written/oral) or the reason that consent was not obtained (e.g. the data were analyzed anonymously)<br><br>**Animal Research (involving vertebrate animals, embryos or tissues)**<br>• Provide the name of the Institutional Animal Care and Use Committee (IACUC) or other relevant ethics board that reviewed the study protocol, and indicate whether they approved this research or granted a formal waiver of ethical approval<br>• Include an approval number if one was obtained<br>• If the study involved *non-human primates*, add *additional details* about animal welfare and steps taken to ameliorate suffering<br>• If anesthesia, euthanasia, or any kind of animal sacrifice is part of the study, include briefly which substances and/or methods were applied<br><br>**Field Research**<br><br>Include the following details if this study involves the collection of plant, animal, or other materials from a natural setting:<br>• Field permit number<br>• Name of the institution or relevant body that granted permission	
**Data Availability**<br><br>Authors are required to make all data underlying the findings described fully available, without restriction, and from the time of publication. PLOS allows rare exceptions to address legal and ethical concerns. See the PLOS Data Policy and FAQ for detailed information.	Yes - all data are fully available without restriction

A Data Availability Statement describing where the data can be found is required at submission. Your answers to this question constitute the Data Availability Statement and **will be published in the article**, if accepted.<br><br>**Important:** Stating 'data available on request from the author' is not sufficient. If your data are only available upon request, select 'No' for the first question and explain your exceptional situation in the text box.<br><br>Do the authors confirm that all data underlying the findings described in their manuscript are fully available without restriction?	
**Describe where the data may be found in full sentences. If you are copying our sample text, replace any instances of XXX with the appropriate details.**<br><br>• If the data are **held or will be held in a public repository**, include URLs, accession numbers or DOIs. If this information will only be available after acceptance, indicate this by ticking the box below. For example: *All XXX files are available from the XXX database (accession number(s) XXX, XXX.).*<br>• If the data are all contained **within the manuscript and/or Supporting Information files**, enter the following: *All relevant data are within the manuscript and its Supporting Information files.*<br>• If neither of these applies but you are able to provide **details of access elsewhere**, with or without limitations, please do so. For example:<br><br>*Data cannot be shared publicly because of [XXX]. Data are available from the XXX Institutional Data Access / Ethics Committee (contact via XXX) for researchers who meet the criteria for access to confidential data.*<br><br>*The data underlying the results presented in the study are available from (include the name of the third party*	All relevant data are within the manuscript and its Supporting Information files

and contact information or URL).<br>• This text is appropriate if the data are owned by a third party and authors do not have permission to share the data.<br><br>* typeset	
Additional data availability information:	

Manuscript Click here to download Manuscript DBM_PLOS.docx

Concurrent of stunting and overweight/obesity among children: evidence from Ethiopia

Alinoor Mohamed Farah[1]

Email: alinuriana@yahoo.com

Tahir Yousuf Nour

Email: daahir571@gmail.com[1]

Bilal Shikur Endris[2]

Email: lebiluka@yahoo.com

Seifu Hagos Gebreyesus[2]

Email: Seif_h23@yahoo.com

Authors' Information:

*[1]Corresponding Author

*[1]Correspondence and reprint requests should be addressed to:

Alinoor Mohamed Farah

College of Medicine and Health Sciences,

Jigjiga University, Jigjiga, Ethiopia

Lecturer at Jigjiga University

Phone: +251911053913

Email: alinuriana@yahoo.com

[1] Department of Public Health, College of Medicine and Health Sciences, Jigjiga University, Ethiopia

[2]Department of Reproductive Health and Health Service Management, School of Public Health, College of Health Sciences, Addis Ababa University, Ethiopia

Abstract

Background: Nutrition transition in many low- and middle-income countries (LMICs) has led to childhood nutritional outcomes to shift from a predominance of undernutrition to a dual burden of under- and overnutrition. Yet, Infant and young child feeding programs in Ethiopia mainly focus on undernutrition. It is therefore crucial to assess the prevalence and determinants to better inform infant young child feeding programs.

Methods: We analyzed anthropometric, sociodemographic and dietary data of children aged 6-23 months from 2016 Ethiopian Demographic and Health Survey (EDHS). A total of 2,674 children were included in the current study. Concurrent of Stunting and Overweight/Obesity (CSO) prevalence was estimated by distal, intermediate and proximal factors. To identify factors associated with CSO, we conducted hierarchical logistic regression analyses.

Results: The overall prevalence of CSO was 2.45%. The odds of CSO was significantly higher in children of low household wealth category, compared to their counter parts in the richest category (AOR=2.07, 95%CI=1.06–4.03, P=0.033). In boys, the likelihood

of CSO was 1.60 times that of girls (95%CI =1.03–2.49, P =0.038). Above 12months of age was significantly associated with a higher odd of CSO, compared to below 12months of age (AOR=1.76, 95% CI=1.07– 2.88, P=0.026).

Small birth size was significantly associated with a higher odd of CSO, compared to large birth size (AOR=2.53, 95%CI=1.45–4.41, P=0.001). Children who did not take vitamin A supplement within the previous six months had a higher odd of CSO, compared to those who took (AOR=1.91, 95%CI =1.19–3.07, P =0.007).

Conclusion: Our study provided evidence on the co-existence of stunting and overweight/obesity among infants and young children in Ethiopia. CSO was associated with various factors originating from community and child levels. Therefore, identifying children at risk of growth flattering and excess weight gain provides IYCF programs in Ethiopia and beyond with an opportunity of earlier interventions.

Key words: Young children, Concurrent of Stunting and Overweight/Obesity, Stunting, Overweight, DHS, Ethiopia

Introduction

Child malnutrition which includes both undernutrition and overweight are global challenges which is associated with an increased risk of mortality and morbidity, unhealthy development, and loss of productivity[1]. Although improvement of child undernutrition in Ethiopia has been achieved, stunting remains an important problem in Ethiopia, with thirty eight percent of children <5 years of age affected. However, economic growth and urbanization in countries like Ethiopia have given rise to a nutrition transition, where there is as shift from traditional diets to “western diets” (energy-dense diets)[2]. This has led to an increase in overweight and obesity. On the other hand, undernutrition decreased but the decline has not been happening as quickly as the rise in overweight and obesity, leading to a double burden of overnutrition and undernutrition[3].

Dual burden of malnutrition could occur at country, household, or individual levels. At household level at least one member is undernourished and at least one member is overweight whereas at individual level the dual burden of malnutrition often manifests as stunting or micronutrient deficiencies co-occurring with overweight or obesity[3-5]. At individual level, history of stunting coupled with consumption of high dense energy foods and micronutrient deficiencies owing to shared underlying

determinants or physiologic links may also result in clustering of nutrition problems such as co-occurrence of stunting and overweight particularly among young children[3].

Children who are stunted and gain abnormal weight later can be at greater risk of unhealthy development than other children. In other words they are at high risk of non-communicable disease since they impose a high metabolic load on a depleted capacity for homoeostasis[6]. Further, concurrent under and over nutrition it a public health challenge in a sense there is a need to strike a delicate balance between reducing undernutrition and preventing over nutrition [5]. There are substantial studies on dual burden of malnutrition and mainly focused on prevalence and trends of double burden at different levels[2-5, 7-28] but few studies investigated factors associated with the dual burden at individual level and in particular among infant and young children [13, 18, 21, 22, 25].

To the best of our knowledge there were no previous studies in Ethiopia on cooccurrence of stunting and overweight among infants and young children that explored factors associated with it. The previous studies focused on inclusive measure of child undernutrition and failed to capture the severity of malnutrition for some children who suffer from more than one type of malnutrition. Therefore, aim of this study is to assess the prevalence of co-

occurrence of stunting and overweight/obese among infants and young children in Ethiopia and factors associated with it.

Study subjects and Methods

Study subjects

We analyzed anthropometric data of children aged 6-23 months from 2016 EDHS which is nationally representative survey. A total of 2925 children aged 6–23 months were found in the dataset. A total of 2674 children were extracted and included in the final dataset after exclusion of incomplete data.

A multistage stratified two- stage cluster sampling procedure was used to select samples. A detailed information of sample design and procedure is presented elsewhere [29]. We included children aged 6-23 months with complete height, weight, dietary and non-dietary data.

Study design and data source

We used data set from national representative cross-sectional household survey that was conducted from September to December 2015. At individual level, we estimated the prevalence of double burden of malnutrition defined as co-existence of stunting and overweight/obese within the same child. Stunting was

defined as length-for-age Z-score (LAZ) below -2SD and overweight/overweight was defined as BMI-for-age Z-score (BAZ) above 2SD from the respective WHO 2006 growth standards reference median. Weight were measured using SECA scales while length was measured using Shorr measuring boards and children were measured while lying down [29].

The data used for this study was extracted from the fourth EDHS. The survey was implemented by Central Statistical Agency (CSA) at the request of Ethiopian Federal Ministry of Health (FMoH) and funded by United States Agency for International Development (USAID).

Data analysis

Children recode data file in the form of STATA was used for analysis. Statistical analysis was performed using the STATA software package, version 14.1 (Stata Corp., College Station, TX, USA). Survey command (svy) was used to adjust for the complex sample design.

We estimated weighted prevalence of CSO by distal, intermediate and proximal factors. Overall differences across the categories were statistically tested using design-based Pearson chi-squared test. Multiple hierarchical logistic regression was used to examine the effect of distal, intermediate and proximal factors. First

bivariate analyses were done for all potential predictors of CSO. Then, hierarchical regression models were run using variables which demonstrated $P<0.20$ during the bivariate analyses. The three-level hierarchical regression models were run following the recommendation of a previous study that suggested to take into account complex hierarchical relationships of different determinants at different level [30]. The first, second, and third models included distal, intermediate, and proximal factors, respectively. As mentioned before, variables with $P<0.20$ value during the bivariate analyses were included in the multiple hierarchical logistic regression analyses. In other words, model-1, 2 and 3 included the distal, intermediate and proximal factors which demonstrated $P<0.20$ during the bivariate analyses. To put it differently, we used model-1 to assess the overall effect of distal factors and excluded the intermediate and proximal factors. We used model 2 to assess the effect of intermediate factors in the presence of distal factors which were considered confounding factors. Proximal factors were entered in model 3 in the presence of distal and intermediate factors which were also considered confounding variables in model 3. Variable significant at p-value of 0.05 during the hierarchical regression analyses was considered to be determinant factor at each model in which the variable was first entered regardless of its performance in the subsequent

model(s). The approach was meant to avoid the possibility of underestimating the effects of distal factors[30].

Result

Background characteristics

The background characteristics of children are presented in Table 1. Fifty three percent of children were females and the rest were male. Sixty six percent of children were older than twelve months and the rest were younger than twelve months. Overall, 31.4 and 11.9% of children were stunted and overweight, respectively. The overall prevalence of CSO was 2.45%. CSO prevalence among urban and rural children was 1.62 and 2.56%, respectively. Among boys and girls, the prevalence was 2.96 and 2.0%, respectively. The age-specific estimates were 1.86 and 2.77% in those aged under 12 months and above 12 months, respectively. The prevalence of CSO by other child characteristics is shown in Table 1.

Table 1: Bivariate analysis of the relation of distal, intermediate, and proximal factors with CSO

		Weighted frequency (%)	CSO prevalence (95% CI)	*P-value**
Basic factors (distal)<br>Residence place	Urban	11.6	1.62 [0.41, 6.14]	0.52
	Rural	88.4	2.56[1.78, 3.67]	
Wealth	Low	44.3	3.28[2.08, 5.14]	0.27
	Middle	22.9	1.94 [0.77, 4.81]	
	High	32.8	1.67[0.79, 3.52]	
Caregiver education	No	62.2	3.31[2.23, 4.89]	0.02
	Primary	31.1	0.68[0.26, 1.78]	
	Secondary+	6.7	2.64[0.63, 10.34]	
Underlying factors (intermediate)				
Water source type	Not improved	42.1	2.26[1.3, 3.88]	0.72
	Improved	57.9	2.59[1.61, 4.15]	
Toilet facility source	Not improved	90.6	2.34[1.64, 3.33]	0.56
	Improved	9.4	3.48[0.95, 11.94]	
Immediate factors (proximal)				
Child sex	Boy	46.9	2.96[1.82, 4.78]	0.30
	Girl	53.1	2.00[1.16, 3.41]	
Child age	<12 months	34.4	1.83[0.81, 4.08]	0.37
	>12 months	65.6	2.77[1.86, 4.11]	
Birth size	Large	31.4	1.86[0.81, 4.22]	0.07
	Average	40.8	1.71[0.87, 3.34]	
	Small	27.8	4.25[2.26, 6.99]	
Infection history	No	62.6	2.12[1.27,3.50]	0.35
	Yes	37.4	3.00[1.80.2.95]	
Breastfeeding duration	<12 months	36.0	1.92[0.90, 4.06]	0.41
	>12 months	64.0	2.75[1.85, 4.07]	
Early breastfeeding initiation	No	15.3	4.37[2.22, 8.43]	0.07
	Yes	84.7	2.10[1.36, 3.23]	
Deworming	No	91.2	2.18[1.47, 3.24]	0.09
	Yes	8.80	5.16[2.06, 12.38]	
Vitamin A supplement	No	60.3	2.99[1.98, 4.50]	0.14
	Yes	39.7	1.62[0.79, 3,27]	
Iron supplement	No	92.3	2.57[1.79, 3.68]	0.06
	Yes	7.7	0.96[0.35, 2.63]	
Grains	No	39.1	1.93[1.03, 3.59]	0.35

	Yes	60.9	2.78[1.81, 4.26]	
Fruits and vegetables	No	70.2	2.71[1.82, 4.02]	0.39
	Yes	29.8	1.82[0.81, 4.07]	
Meat	No	91.7	2.66[1.86, 3.78]	<0.001
	Yes	8.3	0.13[0.00025,0.67]	
Diary	No	82.9	2.20[1.44, 3.35]	0.25
	Yes	17.1	3.64[1.75, 7.41]	
Pulses	No	79.8	2.55[1.73, 3.76]	0.61
	Yes	20.2	2.03[0.90, 4.51]	
Eggs	No	83.8	2.50[1.70, 3.66]	0.80
	Yes	16.2	2.18[0.80,5.82]	
MDD	No	87.4	2.49[1.71, 3.62]	0.80
	Yes	12.6	2.14[0.67, 6.49]	
MMF	No	57.9	1.60[0.92, 2.78]	0.03
	Yes	42.1	3.61[2.24, 5.78]	

CSO: Concurrent of stunting and overweight/obesity CI: confidence interval MDD: Minimum Dietary Diversity MFF: Minimum meal frequency * based on chi-square test of association

Determinants of CSO

The multiple hierarchical logistic regression model presented in Table 2 indicated that house hold wealth, child's age and sex, birth size and vitamin A supplementation were significantly associated CSO.

The odds of CSO was significantly higher in children of low household wealth category, compared to their counter parts in the richest category (AOR=2.07, 95%CI=1.06–4.03, P=0.033). In boys, the likelihood of CSO was 1.60 times that of girls (95%CI =1.03–2.49, P =0.038). Above 12months of age was significantly associated with a higher odd of CSO, compared to below 12months of age (AOR=1.76, 95% CI=1.07– 2.88, P=0.026).

Small birth size was significantly associated with a higher odd of CSO, compared to large birth size (AOR=2.53, 95%CI=1.45–4.41, P=0.001). Children who did not take vitamin A supplement within the previous six months had a higher odd of CSO, compared to those who took (AOR=1.91, 95%CI =1.19–3.07, P =0.007)

Table 2: Hierarchical multiple logistic regression analysis of the relation of basic, underlying, and proximal factors with CSO

**Variable**		**Model 1**		**Model 2**		**Model 3**	
		**AOR (95% CI)**	***P****	**AOR (95% CI)**	***P****	**AOR (95% CI)**	***P****
Residence	Urban	Ref	0.29				
	Rural	1.61(0.67-3.88)					
Wealth index	Low	2.07 (1.06-4.03)	0.033				
	Middle	1.19(0.46-2.70)	0.80				
	High	Ref					
Water source type	Not improved			0.83(0.53-1.30)	0.42		
	Improved			Ref			
Toilet facility type	Not improved			1.92(0.75-4.93)	0.18		
Child sex	Girl					Ref	
	Boy					1.63(1.05-2.54)	0.028
Child age	<12 months					Ref	
	>12 months					1.73(1.06-2.83)	0.030
Birth size	Large					Ref	
	Average					1.00(0.54-1.84)	0.89

	Small	2.47(1.41- 4.31)	0.002
Early breastfeeding initiation	No	1.18(0.71- 1.96)	0.52
	Yes	Ref	
Vitamin A supplement	No	1.77(1.11- 2.82)	0.016
	Yes	Ref	
Meat	No	1.67(0.59-4.73)	0.33
	Yes	Ref	
Fruits and vegetables	No	1.21(0.69-2.14)	
	Yes	Ref	0.50

AOR: Adjusted odds ratio CI: Confidence interval *P-value significant when <0.05

Discussion

Using a national representative sample of infants and young children, the current study aimed to determine the prevalence of CSO and its associated factors among infants and young children in Ethiopia. Our study provided evidence that there was co-occurrence of stunting and overweight among infants and young children in Ethiopia. We found CSO associated with factors at different levels. The basic factor associated with higher odds of CSO was being poor. The immediate factors found associated with higher odds of CSO were male sex, age above 12 months, small birth size and no vitamin A supplement use for the last six months. Our results showed high level of stunting among infants and young children, with 31.4% of children being stunted. Overweight was also prevalent, though not as high as stunting. We found that the 2.45% of children concurrently suffered stunting and overweight/obese. There are studies that documented prevalence of co-occurrence of stunting and overweight among under five children in African countries. The prevalence determined by the current study was higher than studies compared to previous study conducted in Ghana that assessed dual burden of malnutrition among children younger than five years [22] but lower than a study conducted in South Africa and Libya that reported prevalence of 18% and 7% respectively among under five children [15, 27].

Similarly, studies from Asia and Latin America countries determined the prevalence of concurrent of stunting and overweigh. The prevalence of this report was within the range of report from Ecuador that reported a prevalence of 2.8%[10], higher than study from Mexico that reported a prevalence of 1%[16] and lower than a prevalence from China that reported 5.06%[21] . A recent study that examined the dual burden of malnutrition among children aged 6-59 months in the Middle-East and North Africa (MENA) and Latin American and Caribbean (LAC) regions also showed a prevalence that ranged from 0.4 to 10.7% in MENA regions and 0.3 to 1.9% in LAC regions [31]. The prevalence of current study is higher than prevalence of almost all LAC regions and within the range in most countries in MENA regions.

We also found several factors associated with CSO. Children of low household wealth were more likely to be concurrently stunted and overweight/obese. Children of low household wealth were more likely to be concurrently stunted and overweight/obese. This could be due to the fact that infant and young child caring practices like hygiene, proper feeding and health services utilization are often poorly practiced among poor households compared to their richer counterparts[32-34]. This finding also agrees with the existing literature on concurrent of stunting and overweight [22, 25]. Children who reside in the rural areas had a higher risk of

being concurrently stunting and overweight though insignificant. This finding was also consistent with the existing literature which shows children residing in rural and less urbanized areas are at risk of being concurrently stunted and overweight[21].

We also found higher odds of CSO in boys and those children above 12 months of age. This could be due to fact that most stunting occurs more during the period 12 to 23months of age[32, 35]. In general, children under-two years of age bear a higher burden of both overweight and stunting, particularly in LMICs countries[21]. Further, our finding of higher risks of CSO in boys than in girls was not in agreement with previous reports which demonstrated higher odds of stunting and overweight in girls [22].

Small birth size was also significantly associated with CSO. This finding was also in agreement with the existing evidence which suggests low birthweight linked to poor health and nutritional outcomes [1, 35, 36]. Fetal growth restriction is an important contributor to stunting in children and evidence showed that low birthweight was associated with 2.5–3.5-fold higher odds of wasting, stunting and underweight[36]. Further, other evidences have suggested low birth weight babies who exhibit catchup growth may be at risk of abnormal weight gain in childhood[37-41]. In other words, intra uterine growth retardation might induce a catch-up fat mass for the fetus before even born [42] and the rapid

weight gain appears to promote adiposity after about age 2 years particularly in LMICs [43]. Thus, it could be easily acknowledged that low birth weight child would be at a higher risk of being concurrently affected by stunting and overweight/obesity. Vitamin A intake was also associated with a significantly lower CSO prevalence. This would be most likely due to the role of vitamin A in promoting linear growth and reduce the risk of stunting[44-47]. The first 1,000 days is a critical time for physical and intellectual growth and set a foundation for long term heath and development [1]. As a result of greater awareness of significance of stunting as one of major public health problems, stunting reduction has gained increased international attention [48]. Similarly, overweight/obesity in this age group deserves attention because at early stage of life catch up growth have been identified as one of the risk factors that lead to progression of abnormal weight gain. [49-55]. Therefore, it is important to identify children that are at risk of developing CSO as early as possible to limit progression of both growth flattering and abnormal weight gain. Further, evidence have shown that if only under nutrition is targeted with an aim of targeting growth may unintentionally contribute to abnormal weight gain[56].

The current study has the following limitations; first, recall bias while reporting the birth, infection, dietary history of children is

still an issue of concern [57]. In other words, collecting data like birth size, history of infection and dietary related data are solely based on memory of mothers or the caretaker which might have led to recall bias. Further, due to the cross-sectional nature of this study a cause and effect relationship could not be inferred.

Given the above-mentioned limitations, the current work has some strengths. First, the EDHS data is a national representative data and conclusions about Ethiopia can be drawn. Second the data is reliable and of high quality since the standardized procedures are employed by such kind of survey. Third, appropriate statistical method was used to explore the relationships between the outcome variable and its determinants.

Conclusion

In conclusion, our study provided evidence on the co-existence of stunting and overweight/obesity among infants and young children in Ethiopia. CSO was associated with various factors originating from community and child levels. Therefore, identifying children at risk of growth flattering and excess weight gain provides IYCF programs in Ethiopia and beyond with an opportunity of earlier interventions.

Abbreviations:

AOR	Adjusted Odds Ratio
BAZ	Body Mass Index for Age Z-score
BMI	Body Mass Index
CI	Confidence Interval
CSO	Concurrent of Stunting and Overweight/Obesity
EA	Enumeration Area
EDHS	Ethiopia Demographic and Health Survey
IYCF	Infant and Young Child Feeding
LAZ	Length for Age Z-score
IRB	Institutional Review Board
LAC	Latin American and Caribbean
LMIC	Lower, Middle Income Countries
MAD	Minimum Acceptable Diet
MENA	Middle-East and North Africa
MDD	Minimum Dietary Diversity
MMF	Minimum Meal Frequency
NRERC	National Research Ethics Review Committee

SGA Small for Gestational Age

UNICEF United Nations Children’s Fund

USAID United States of Agency for International Development

WHO World Health Organization

Declarations

Acknowledgment

The authors will like to thank ICF international to grant permission to use the EDHS data.

Funding

Not applicable

Availability of data and materials

The dataset for this study is available from the corresponding author on reasonable request.

Ethical approval

Ethical clearance for the survey was provided by Institutional Review Board (IRB) of the College of Medicine and Health Sciences at Jigjiga University. Online application to analyze the secondary data was requested from DHS Program, USAID and we

have been authorized to download data from the Demographic and Health Surveys (DHS) online archive.

Consent for publication

Not applicable

Conflict of interest:

The authors declare that they have no competing of interest.

Authorship:

AM conceived the study, prepared the proposal, analyzed the data, interpreted the findings and wrote the manuscript. TY, BS and SH were involved in developing the study proposal, data analysis and reviewing the manuscript.

References

1. Black, R.E., et al., *Maternal and child undernutrition and overweight in low-income and middle-income countries.* The lancet, 2013. **382**(9890): p. 427-451.
2. Popkin, B.M., *The nutrition transition in low-income countries: an emerging crisis.* Nutrition reviews, 1994. **52**(9): p. 285-298.
3. Tzioumis, E., et al., *Prevalence and trends in the childhood dual burden of malnutrition in low-and middle-income countries, 1990–2012.* Public health nutrition, 2016. **19**(8): p. 1375-1388.
4. Doak, C.M., et al., *The dual burden household and the nutrition transition paradox.* International journal of obesity, 2005. **29**(1): p. 129.
5. Garrett, J.L. and M.T. Ruel, *Stunted child–overweight mother pairs: prevalence and association with economic development and urbanization.* Food and nutrition bulletin, 2005. **26**(2): p. 209-221.
6. Wells, J.C., et al., *The double burden of malnutrition: aetiological pathways and consequences for health.* The Lancet, 2019.
7. Jinabhai, C.C., M. Taylor, and K.R. Sullivan, *Changing patterns of under-and over-nutrition in South African children—future risks of non-communicable diseases.* Annals of tropical paediatrics, 2005. **25**(1): p. 3-15.
8. Cattaneo, A., et al., *Child nutrition in countries of the Commonwealth of Independent States: time to redirect strategies?* Public health nutrition, 2008. **11**(12): p. 1209-1219.
9. Ferreira, H.d.S. and S.C.M. Luciano, *Prevalence of extreme anthropometric measurements in children from Alagoas, Northeastern Brazil.* Revista de saude publica, 2010. **44**(2): p. 377-380.
10. Freire, W.B., et al., *The double burden of undernutrition and excess body weight in Ecuador.* The American journal of clinical nutrition, 2014. **100**(6): p. 1636S-1643S.
11. Gardner, K., et al., *Prevalence of overweight, obesity and underweight among 5‐ year‐ old children in Saint Lucia by three methods of classification and a comparison with historical rates.* Child: Care, Health and Development, 2011. **37**(1): p. 143-149.
12. Jafar, T., et al., *Rise in childhood obesity with persistently high rates of undernutrition among urban school-aged Indo-Asian children.* Archives of disease in childhood, 2008. **93**(5): p. 373-378.

13. Kavle, J.A., et al., *Factors associated with early growth in E gyptian infants: implications for addressing the dual burden of malnutrition.* Maternal & child nutrition, 2016. **12**(1): p. 139-151.
14. Khor, G.L. and Z.M. Sharif, *Dual forms of malnutrition in the same households in Malaysia--a case study among Malay rural households.* Asia Pacific Journal of Clinical Nutrition, 2003. **12**(4).
15. Kimani-Murage, E.W., et al., *The prevalence of stunting, overweight and obesity, and metabolic disease risk in rural South African children.* BMC public health, 2010. **10**(1): p. 158.
16. Kroker-Lobos, M.F., et al., *The double burden of undernutrition and excess body weight in Mexico.* The American journal of clinical nutrition, 2014. **100**(6): p. 1652S-1658S.
17. Motlagh, M.E., et al., *Double burden of nutritional disorders in young Iranian children: findings of a nationwide screening survey.* Public health nutrition, 2011. **14**(4): p. 605-610.
18. Saibul, N., et al., *Food variety score is associated with dual burden of malnutrition in Orang Asli (Malaysian indigenous peoples) households: implications for health promotion.* Asia Pacific journal of clinical nutrition, 2009. **18**(3): p. 412-422.
19. Sarmiento, O.L., et al., *The dual burden of malnutrition in Colombia.* The American journal of clinical nutrition, 2014. **100**(6): p. 1628S-1635S.
20. Wang, X., et al., *Stunting and 'overweight'in the WHO Child Growth Standards–malnutrition among children in a poor area of China.* Public health nutrition, 2009. **12**(11): p. 1991-1998.
21. Zhang, N., L. Bécares, and T. Chandola, *Patterns and determinants of double-burden of malnutrition among rural children: evidence from China.* PloS one, 2016. **11**(7): p. e0158119.
22. Atsu, B.K., C. Guure, and A.K. Laar, *Determinants of overweight with concurrent stunting among Ghanaian children.* BMC pediatrics, 2017. **17**(1): p. 177.
23. Provo, A., *Towards Sustainable Nutrition for All Tackling the double burden of malnutrition in Africa.* Sight Life, 2013. **27**(3): p. 40-7.
24. Urke, H.B., M.B. Mittelmark, and M. Valdivia, *Trends in stunting and overweight in Peruvian pre-schoolers from 1991 to 2011: findings from the Demographic and Health*

Surveys. Public health nutrition, 2014. **17**(11): p. 2407-2418.

25. Fernald, L.C. and L.M. Neufeld, *Overweight with concurrent stunting in very young children from rural Mexico: prevalence and associated factors.* European journal of clinical nutrition, 2007. **61**(5): p. 623.
26. Said-Mohamed, R., et al., *Determinants of overweight associated with stunting in preschool children of Yaounde, Cameroon.* Annals of human biology, 2009. **36**(2): p. 146-161.
27. Adel, E.T., et al., *Nutritional status of under-five children in Libya; a national population-based survey.* Libyan Journal of Medicine, 2008. **3**(1): p. 13-19.
28. Hassen, K., G. Gizaw, and T. Belachew, *Dual burden of malnutrition among adolescents of smallholder coffee farming households of Jimma zone, Southwest Ethiopia.* Food and nutrition bulletin, 2017. **38**(2): p. 196-208.
29. CSA, I., *Central statistical agency (CSA)[Ethiopia] and ICF.* Ethiopia demographic and health survey, Addis Ababa, Ethiopia and Calverton, Maryland, USA, 2016.
30. Victora, C.G., et al., *The role of conceptual frameworks in epidemiological analysis: a hierarchical approach.* International journal of epidemiology, 1997. **26**(1): p. 224-227.
31. Ghattas, H., et al., *Child‐ level double burden of malnutrition in the MENA and LAC regions—Prevalence and social determinants.* Maternal & Child Nutrition, 2019.
32. Victora, C.G., et al., *Worldwide timing of growth faltering: revisiting implications for interventions.* Pediatrics, 2010. **125**(3): p. e473-e480.
33. Melaku, Y.A., et al., *Associations of childhood, maternal and household dietary patterns with childhood stunting in Ethiopia: proposing an alternative and plausible dietary analysis method to dietary diversity scores.* Nutrition journal, 2018. **17**(1): p. 14.
34. Farah, A.M., B.S. Endris, and S.H. Gebreyesus, *Maternal undernutrition as proxy indicators of their offspring's undernutrition: evidence from 2011 Ethiopia demographic and health survey.* BMC Nutrition, 2019. **5**(1): p. 17.
35. Danaei, G., et al., *Risk factors for childhood stunting in 137 developing countries: a comparative risk assessment analysis at global, regional, and country levels.* PLoS medicine, 2016. **13**(11): p. e1002164.
36. Christian, P., et al., *Risk of childhood undernutrition related to small-for-gestational age and preterm birth in*

low-and middle-income countries. International journal of epidemiology, 2013. **42**(5): p. 1340-1355.

37. Kuhle, S., et al., *Birth weight for gestational age, anthropometric measures, and cardiovascular disease markers in children.* The Journal of pediatrics, 2017. **182**: p. 99-106.
38. Gallo, P., et al., *SGA children in pediatric primary care: what is the best choice, large or small? A 10-year prospective longitudinal study.* Global pediatric health, 2016. **3**: p. 2333794X16659993.
39. Yu, Z., et al., *Birth weight and subsequent risk of obesity: a systematic review and meta‐ analysis.* Obesity Reviews, 2011. **12**(7): p. 525-542.
40. Kramer, M.S., et al., *Is restricted fetal growth associated with later adiposity? Observational analysis of a randomized trial.* The American journal of clinical nutrition, 2014. **100**(1): p. 176-181.
41. Ong, K.K., et al., *Association between postnatal catch-up growth and obesity in childhood: prospective cohort study.* Bmj, 2000. **320**(7240): p. 967-971.
42. Hemachandra, A.H. and M.A. Klebanoff, *Use of serial ultrasound to identify periods of fetal growth restriction in relation to neonatal anthropometry.* American Journal of Human Biology: The Official Journal of the Human Biology Association, 2006. **18**(6): p. 791-797.
43. Adair, L.S., et al., *Associations of linear growth and relative weight gain during early life with adult health and human capital in countries of low and middle income: findings from five birth cohort studies.* The Lancet, 2013. **382**(9891): p. 525-534.
44. Permeisih, D., Y. Idjradinata, and D. Karyadi, *Vitamin A-fortified monosodium glutamate and health, growth, and survival of children: a controlled field trial.* The American journal of clinical nutrition, 1988. **48**(5): p. 1271-1276.
45. Organization, W.H., *Essential nutrition actions: improving maternal, newborn, infant and young child health and nutrition.* 2013.
46. Mwanri, L., et al., *Supplemental vitamin A improves anemia and growth in anemic school children in Tanzania.* The Journal of nutrition, 2000. **130**(11): p. 2691-2696.
47. Semba, R.D., et al., *Low intake of vitamin A–rich foods among children, aged 12–35 months, in India: association with malnutrition, anemia, and missed child survival interventions.* Nutrition, 2010. **26**(10): p. 958-962.
48. Aguayo, V.M. and P. Menon, *Stop stunting: Improving child feeding, women's nutrition and household sanitation*

in South Asia. Maternal & child nutrition, 2016. **12**: p. 3-11.
49. Okihiro, M., et al., *Rapid growth from 12 to 23 months of life predicts obesity in a population of Pacific Island children.* Ethnicity & disease, 2012. **22**(4): p. 439.
50. Chomtho, S., et al., *Infant growth and later body composition: evidence from the 4-component model.* The American journal of clinical nutrition, 2008. **87**(6): p. 1776-1784.
51. Dennison, B.A., et al., *Rapid infant weight gain predicts childhood overweight.* Obesity, 2006. **14**(3): p. 491-499.
52. Druet, C., et al., *Prediction of childhood obesity by infancy weight gain: an individual‐ level meta‐ analysis.* Paediatric and perinatal epidemiology, 2012. **26**(1): p. 19-26.
53. Taveras, E.M., et al., *Weight status in the first 6 months of life and obesity at 3 years of age.* Pediatrics, 2009. **123**(4): p. 1177-1183.
54. Monteiro, P.O.A. and C.G. Victora, *Rapid growth in infancy and childhood and obesity in later life–a systematic review.* Obesity reviews, 2005. **6**(2): p. 143-154.
55. Slining, M., et al., *Infant BMI trajectories are associated with young adult body composition.* Journal of developmental origins of health and disease, 2013. **4**(1): p. 56-68.
56. Victora, C.G. and F.C. Barros, *Commentary: the catch-up dilemma—relevance of Leitch's 'low–high'pig to child growth in developing countries.* International journal of epidemiology, 2001. **30**(2): p. 217-220.
57. Ties Boerma, J. and A.E. Sommerfelt, *Demographic and health surveys (DHS: contributions and limitations.* 1993.
