# Supplementary material for: Concurrence of stunting and overweight/obesity among children: Evidence from Ethiopia
Source: PLoS One. 2021 Jan 15;16(1):e0245456. doi: 10.1371/journal.pone.0245456 (PMC7810347; doi:10.1371/journal.pone.0245456)
Supplement: Attachment — Submitted filename: Comments to the Editor.docx [file pone.0245456.s001.docx]

**Comments to Editor**

Dear Editor,

Thank you very much for the opportunity to review the manuscript looking into the co-occurrence of stunting and overweight in Ethiopian children. In my eyes the context and the ideas of the paper are very relevant, especially giving the available data set. However, the authors need to revise the language to improve readability, some sections were very hard to follow and understand. Further, a revision of the layout of the paper could help to better review and contribute to the work (e.g. missing page numbers, use of different paragraphs). Not clear to me is that an ethical statement was mentioned at the end of the manuscript, but in the respective section in front of the paper it is not mentioned.

Kind regards, Andrea Fongar
