# Supplementary material for: Concurrence of stunting and overweight/obesity among children: Evidence from Ethiopia
Source: PLoS One. 2021 Jan 15;16(1):e0245456. doi: 10.1371/journal.pone.0245456 (PMC7810347; doi:10.1371/journal.pone.0245456)
Supplement: Attachment — Submitted filename: Response_to_reviewers.docx [file pone.0245456.s004.docx]

**Reviewer comments**

Title: Concurrent of stunting and overweight/obesity among children: evidence from Ethiopia

Date: Sep 17 2020

Dear Editor,

We would like to thank you and the reviewers for the comments provided to our manuscript which help to improve the quality of our manuscript. Our response is as follows:

| **Reviewers Comments** | **Author’s Revision and Response** |
| --- | --- |
| Reviewer 1 |  |
| Thanks to the authors for the extensive revision of the manuscript, while most of the manuscript has improved, I remain with further comments to the authors for clarification of the manuscript. Additionally, I believe the manuscript could further benefit from language edition to improve readability. First, I want to mention that my line references are to the manuscript without track changes (the first one within the PDF). | - We would like to thank reviewer for the positive appraisal on the revised work. - Language improved. Dr. Olusola Oladeji has edited the manuscript. |
| Thank you for mentioning upfront the shift of focus of the age bracket of the children from 6-23 towards 6-59 months of age. However, could the authors please elaborate further on why the shift was done. | - The shift of focus from age group of 6-23 to 6-59 months was mainly for generalizability. Besides, most of the literatures cited in our work focused on 6-59 months. |
| The authors mentioned dietary data availability as one point to expand the age bracket. Within the discussion dietary effects are used to explain the difference between agrarian and pastoralists. The available dietary data for children 6-23 month, if analyzed could strengthen this point (Line 476 ff) as a consistency check. I believe these results were mentioned in the previous version of the manuscript and could be included as a footnote for the sub-sample. | - Agreed, and we would like to thank the reviewer for this valuable comment. Sub sample analysis done for data of children aged 6-23 months and found that 62% and only 15% of children from pastoralist and agrarian communities respectively consumed dairy products (P<0.001) * - * Pearson chi-squared test |
| While the manuscript improved substantially, I still would advise to revision the manuscript further for readability and uniformity. Different writing styles are used within the manuscript (cooccurrence vs co-occurrence (line 77 vs. 108), presentation of results numeric and verbal (line 84 vs 92). Additionally, have a keen eye on the style of literature which is inconsistent. While the authors tried to in cooperate the comment on the naming of the double burden of malnutrition please re-check for consistency | - Agreed, consistency rechecked and “concurrence” used throughout the manuscript. - Agreed, numeric used consistently throughout the manuscript - Consistency of literature rechecked |
| Within the Abstract the authors mention in line 55 ‘younger than 12 months. Within the conclusion ‘younger than 12 years of age’ is mentioned (line 601 f). Please revise. | - Agreed, revised and replaced with younger than 5 years. |
| I appreciate the effort and work the authors put into rewriting the method section as requested by the reviewers, while the new structure is more intuitive, some information is in my eyes still explained within the wrong section. The first section study design and data source still fail to name the data set, the name (EDHS line 188) are introduced within the next section study subjects. I, therefore, advise revising the section making sure the present information is fitting to the current section naming. | - Agreed, section revised |
| Thanks to the authors to include further information on the selection of the children. I still would like to get more clarification on the sample size construction. The authors mentioned children not alive (lien 184 ff and figure 1) were dropped for the analysis. Please elaborate on how this data was collected or even present within the data set. | - DHS surveys use a full birth history which is a complete list of all children a mother has ever given birth to including their date of birth, sex, survival status, age (if alive), and age at death (if died). This is the form of birth history found in the majority of DHS surveys. |
| While the authors took up the suggestion to remain with one name of the categorization of basic, underlying and immediate factors, within the manuscript the previous names are still present. Please revised to be consistent throughout the manuscript (see as example line 331).  Line 251ff sentence is repetitive please review. | - Agreed, basic, underlying and immediate factors were used consistently throughout the manuscript. |
| Thanks for the inclusion of Table 1, which displays information about the associated factors. However, the Table would benefit from revision be improved readability and visibility for the reader. Please remove all the information not necessary to the construction of the variable. For example, CSO only needs the definition applied to the dummy variable. Please revise the Table numbering and layout. Most tables have several table numbers listed. | - Agreed, unnecessary information to construct the variables removed and table layout and numbering revised. |
| Sentence in line 432 is not clear to me, please revise. | - Agreed and revised as follows: “The immediate factors found associated with higher odds of CSO were age younger than 12 months, no history of infection and not having received deworming for the last six months”. |
