# Supplementary material for: Concurrence of stunting and overweight/obesity among children: Evidence from Ethiopia
Source: PLoS One. 2021 Jan 15;16(1):e0245456. doi: 10.1371/journal.pone.0245456 (PMC7810347; doi:10.1371/journal.pone.0245456)
Supplement: Attachment — Submitted filename: 3rd_Response_to_reviewers.docx [file pone.0245456.s005.docx]

**Reviewer comments**

Title: Concurrence of stunting and overweight/obesity among children: evidence from Ethiopia

Date: November 04, 2020

Dear Editor,

We would like to thank you and the reviewer for the comments provided to our manuscript which help to improve the quality of our manuscript. Our response is as follows:

| **Reviewers Comments** | **Author’s Revision and Response** |
| --- | --- |
| Reviewer 1 |  |
| The manuscript has improved language-wise, still please have a keen eye on the writing style age of the children (for example line 83 vs. line 109 or line 134 or line 615f). | We would like to thank the reviewer for point this out. “children under five years of age” was consistently used through out the manuscript. |
| Please revise the following sentences as they hold repetitive information: line 87f. | Revised and the repetitions removed |
| Please revise the sentence in line 137ff. which is unclear to me. | Revised as follows: “Children who are concurrently stunted and overweight/obese can be at greater risk of unhealthy development than normal children” |
| Thanks for the revision at the study subject area, I appreciate the idea of a figure, though I was not able to allocate the figure for the review. Please make sure to include the figure in the attachments. | Figure attached |
| Please check line 343 the table has two numbers. | Repetition removed |
|  |  |
